# Spectral expansion methods for prediction uncertainty quantification in systems biology

**DOI:** 10.3389/fsysb.2024.1419809

**Published:** 2024-10-03

**Authors:** Anna Deneer, Jaap Molenaar, Christian Fleck

**Affiliations:** ^1^ Mathematical and Statistical Methods Group, Wageningen University and Research, Wageningen, Netherlands; ^2^ Freiburg Center for Data Analysis and Modeling, University of Freiburg, Freiburg, Germany

**Keywords:** systems biology, computational systems biology, mathematical modelling, spectral expansion, surrogate models

## Abstract

Uncertainty is ubiquitous in biological systems. For example, since gene expression is intrinsically governed by noise, nature shows a fascinating degree of variability. If we want to use a model to predict the behaviour of such an intrinsically stochastic system, we have to cope with the fact that the model parameters are never exactly known, but vary according to some distribution. A key question is then to determine how the uncertainties in the parameters affect the model outcome. Knowing the latter uncertainties is crucial when a model is used for, e.g., experimental design, optimisation, or decision-making. To establish how parameter and model prediction uncertainties are related, Monte Carlo approaches could be used. Then, the model is evaluated for a huge number of parameters sets, drawn from the multivariate parameter distribution. However, when model solutions are computationally expensive this approach is intractable. To overcome this problem, so-called spectral expansion (SE) methods have been developed to quantify prediction uncertainty within a probabilistic framework. Such SE methods have a basis in, e.g., computational mathematics, engineering, physics, and fluid dynamics, and, to a lesser extent, systems biology. The computational costs of SE schemes mainly stem from the calculation of the expansion coefficients. Furthermore, SE effectively leads to a surrogate model which captures the dependence of the model on the uncertainty parameters, but is much simpler to execute compared to the original model. In this paper, we present an innovative scheme for the calculation of the expansion coefficients. It guarantees that the model has to be evaluated only a restricted number of times. Especially for models of high complexity this may be a huge computational advantage. By applying the scheme to a variety of examples we show its power, especially in challenging situations where solutions slowly converge due to high computational costs, bifurcations, and discontinuities.

## 1 Introduction

Every mathematical model in systems biology is subject to uncertainty and incomplete knowledge (Geris and Gomez-Cabrero, 2016; Gutenkunst et al., 2007; Mitra and Hlavacek, 2019; van Mourik et al., 2014). This can be in the form of unknown model structure, unknown model parameters and imperfect experimental data. Characterizing and quantifying these sources is crucial, as the uncertainty can translate into inaccuracies in the model predictions. Information about the quality of model predictions is vital when applied as support for decision-making or optimization routines such as experimental design and parameter estimation (Raue et al., 2013). The aim of uncertainty quantification (UQ) is to determine the likeliness of certain outcomes, given that some aspects of the system under study are not (exactly) known.

Generally, uncertainty is distinguished into two classes (Bruno, 2007; Ghanem et al., 2017; Le Maître and Knio, 2010). The first class is so-called aleatoric uncertainty. Aleatoric uncertainty stems from the intrinsic variability found in the system under consideration, for this reason it is also referred to as statistical uncertainty. For example, in the case of parameter estimation, this uncertainty is related to the fact that parameters may essentially vary over the system components (e.g., cells) (Elowitz et al., 2002), so that for the system as a whole only a distribution of parameter values can be estimated, and not one precise value per parameter. Gene expression noise is often a source of varying conditions (e.g., initial conditions or protein production rates) in cells resulting in variability of process parameters such as the protein production rate (Thattai and van Oudenaarden, 2001; Elowitz et al., 2002; Blake et al., 2003; Lev, 2014; Schultheiß Araújo et al., 2017).

In contrast, the second class of uncertainty, termed epistemic (or systemic) uncertainty, is caused by a lack of information (Bruno, 2007; Ghanem et al., 2017; Le Maître and Knio, 2010). For example, in parameter estimation, uncertainty in the estimates may be caused by imperfect data sets that contain noisy, incoherent, or even missing data points (Sullivan, 2015). In such cases, the uncertainty can in principle be reduced by performing extra experiments (Banga and Balsa-Canto, 2008; Kreutz and Timmer, 2009; Barz et al., 2010), but practical restrictions often prohibit this.

In biological systems both types of uncertainty are typically present (Geris and Gomez-Cabrero, 2016). In terms of modelling, both are usually dealt with by employing a probabilistic framework (Kirk et al., 2016), in which model parameters are represented according to a probability density function (PDF) (van Mourik et al., 2014; Bruno, 2007). The choice of the type of PDF and the corresponding distribution of parameters is usually based on previous knowledge. For example, the case of a completely unknown parameter is described by a uniform distribution on a broad (positive) interval. In other cases a parameter could be known to follow a normal or lognormal distribution with known mean and variance, established in previously performed experiments (Tsigkinopoulou et al., 2018).

Among the field of UQ, Monte Carlo (MC) methods are most commonly used (Barbu and Zhu, 2020; James, 1980). In an MC approach the parameter PDFs are sampled and model responses for each sample recorded, thus providing a distribution of model outcomes and an indication of the uncertainty therein (e.g., by analyzing the distribution moments). These methods are simple in their implementation and are widely applicable. However, for models that have a large number of parameters or are computationally expensive, these MC procedures are often not feasible (Bruno, 2007; Le Maître and Knio, 2010).

As an alternative to MC, meta-modelling techniques, also referred to as surrogate modelling, are frequently adopted to deal with models that would otherwise be intractable. Support vector machines (Noble, 2004), artificial neural networks (Tadeusiewicz, 2015) and Bayesian networks (Wilkinson, 2007) are examples meta-modelling techniques used in systems biology. In this work, we focus on spectral expansion (SE) methods, an approach that is widely used in engineering systems (Ghanem et al., 2017; Le Maître and Knio, 2010) and to a lesser extent in biological systems (Martin-Casas and Mesbah, 2016; Paulson et al., 2019; Streif et al., 2014; Renardy et al., 2018) for UQ purposes. In such an approach the model response is represented in the form of a series expansion. The advantage of such a representation is that an approximation of the model response is obtained for *all* values of the parameters at once. This allows immediate evaluation of statistics of the model outcome, either analytically or through sampling of the stochastic parameters, which can be done significantly faster than through MC methods for models that are problematic and computationally expensive (Xiu and George, 2002).

These advantages come at the cost of the need to calculate expansion coefficients. For this, two classes of spectral methods are in use. In the first class the governing equations of the model are reformulated such that each variable is represented by a spectral expansion. This results in a system of differential equations for the expansion coefficients and is known as intrusive spectral projection (Bruno, 2007). In the second class, based on so-called non-intrusive spectral projection, the expansion coefficients are determined without changing the original model equations (Eldred et al., 2008). This is the line followed in this paper. The advantage of a non-intrusive approach is that it requires only straightforward deterministic model evaluations and does not involve any reformulation of the model. It is particularly attractive in case of models where intrusive methods would become too laborious.

In the past, SE methods have been shown to converge very slowly or not at all for models involving non-smooth functions (Joel Chorin, 1974; Le Maıtre et al., 2004). This is indeed a critical challenge for biological models, which often show complex, non-linear behaviour such as bifurcations and spatial discontinuities. In this work, we provide a scheme for non-intrusive spectral projection that may overcome these problems. It is easy to implement and we show its power through applying it to a number of various biological models. The scheme straightforwardly allows the use of different basis functions for the expansion, such as Haar wavelets in case of models with bifurcations. We also present a simple segmentation method resulting in a piecewise continuous approximation of the model at hand which helps to deal with complex models avoiding the necessity of high order expansions. The examples treated in this paper have been chosen such that each one shows how a specific problem can be overcome. The MATLAB code used for each example is publicly available on GitHub (see Section 8)

## 2 Methods

### 2.1 Spectral expansion

Let us consider a model Ω that depends on a vector of stochastic input parameters θ . The model response Y can be any chosen quantity, e.g., the concentration of one of the model components or a function thereof. Here, the uncertainty parameters $\theta_{l},\;l=1,\ldots ,U$ , are assumed to be uncorrelated and thus independently distributed, each with PDF $P_{l}\left(\theta_{l}\right)$ . So,

$$Y=\Omega \left(\theta \right),\;\theta ∼P\left(\theta \right),$$ (1)

where P(θ) is the joint probability density function (PDF) for all U uncertainty parameters: $P\left(\theta \right)=\prod_{l=1}^{U}P_{l}\left(\theta_{l}\right)$ . The case of correlated parameters is discussed in Section 2.2.3. For reasons of clarity, we restrict the explanation in this section to the univariate case, i.e., Y and θ , as shown in Equation 1, are scalars. In Section 2.2 we show how to deal with more than one uncertainty parameter.

The underlying model could be of any type, e.g., an ODE, a PDE, an algebraic, or a statistical model. This implies that Y may also depend on time and space. The challenge is to analyse the behaviour of Y as a function of θ . In cases where the numerical evaluation of the underlying model takes a considerable amount of computational time, this tends to obstruct any form of comprehensive analysis. In this paper we present a method that aims at making this tractable. The idea is to replace the original model by a meta-model, which is achieved by representing the output Y in terms of an expansion. This meta-model can be constructed such that it represents the underlying model to a high degree of accuracy, but having the advantage that it is much faster to evaluate than the original model.

This meta-model can be used to determine the distribution of the model response Y or reconstruct the function accurately at given points in the parameter space. In the spectral expansion (SE) approach the model response is represented by

$$Y\left(\theta \right)=\overset{\infty }{\underset{n=0}{\sum }}c_{n}\phi_{n}\left(\theta \right),$$ (2)

where $c_{n}$ are the expansion coefficients (which can be time and/or space dependent) and $\phi_{n}$ are functions that are orthonormal with respect to the distribution of the uncertainty parameters as weight functions for an inner product, as dealt with in Supplementary Material S1. For example, suitable basis functions for uniformly distributed parameters are Legendre polynomials, while for normally distributed parameters Hermite polynomials qualify. For any practical purpose the expansion given in Equation 2 needs to be truncated to a certain degree:

$$Y^{s}\left(\theta \right)=\overset{N}{\underset{n=0}{\sum }}c_{n}\phi_{n}\left(\theta \right),$$ (3)

where N is the truncation degree. For the meta-model $Y^{s}\;N$ expansion coefficients have to be calculated. The advantage of Equation 3 is that the statistics of the model response Y can be evaluated very fast, either analytically or through sampling of the parameters θ . The main computational cost of the SE approach stems from the computation of the coefficients $c_{n}$ . Below, we provide an easy-to-implement scheme for the calculation of these coefficients.

The most commonly used method for determining the coefficients in the SE approach is through Gaussian quadrature schemes (Bruno, 2007). Here, we propose an alternative scheme. It is applicable to any set of orthonormal functions, allowing the flexibility to tackle different modelling challenges. A key feature in the scheme is the introduction of the symmetric matrix:

$${\hat{B}}_{n,m}=\int \phi_{n}\left(\theta \right)\;\theta \;\phi_{m}\left(\theta \right)\;P\left(\theta \right)\;d\theta .$$ (4)

Since this matrix is symmetric, its eigenvalues $\lambda^{\left(l\right)},\;l=1,2,\ldots$ , are real and its eigenvectors $u^{\left(l\right)}$ orthonormal. In the Supplementary Material S1 we show that these eigenvalues and eigenvectors can be used to derive an explicit expression for the coefficients $c_{n}$ . After substitution of this expression, Equation 3 then reads as

$$Y^{s}\left(\theta \right)=\overset{N}{\underset{l=1}{\sum }}Y\left(\lambda^{\left(l\right)}\right)\;u_{1}^{\left(l\right)}\;\psi_{l}^{s}\left(\theta \right),$$ (5)

where

$$\psi_{l}^{s}\left(\theta \right)\equiv \overset{N}{\underset{n=0}{\sum }}u_{n+1}^{\left(l\right)}\;\phi_{n}\left(\theta \right).$$ (6)

The striking point here is that this expansion requires evaluation of the model only N times, namely, for each of the eigenvalues $\lambda^{\left(l\right)},\;l=1,\ldots ,N$ . Note that all terms in the expansion that do not depend on the uncertainty parameter θ can be calculated in advance, so once and for all. This saves computation time for any future application. For models that take a long time to evaluate the use of Equation 5 is a very fast alternative, compared to e.g., a Monte-Carlo approach. What’s more, statistical moments like mean and variance follow directly from the expansion coefficients (Bruno, 2007), thus requiring no further calculation. Note that the expansion in Equation 5 is only exact in the limit N→∞ . Taking a finite value for N introduces inaccuracy. Therefore, N must be chosen with care and it is often not obvious beforehand which value of N will give reliable results. In the examples below we have chosen N by visual comparison with e.g., an analytical solution or with MC solutions. In cases when a high level of accuracy is needed a more robust comparison or measure of convergence will be required, see for example, (Vincent et al., 2013) where a leave-one-out technique is used as a stopping criterion. In Section 2.2.4, we propose an easy-to-use scheme to deal with cases where a high degree of expansion might result in infeasible computation times.

### 2.2 Practical aspects

Here, we treat some specific aspects of the method presented above.

#### 2.2.1 PDFs and basis functions

We already mentioned Legendre and Hermite polynomials as possible basis functions for SE. Legendre polynomials are defined over the interval $\left[-1,1\right]$ and are orthogonal with respect to the uniform distribution U(−1,1) . The Hermite polynomials are defined over the whole real axis R and are orthogonal with respect to the Gaussian distribution N(0,1) , with mean 0 and standard deviation 1. Both polynomials can be normalized with appropriate prefactors to ensure orthonormality. These two families of polynomials are most often used to represent biological parameters. The uniform distribution is typically applied in uninformed cases and the lognormal distribution in cases where there is prior information available on a parameter. In practice, sometimes other classical orthogonal polynomial families are appropriate, e.g., Laguerre polynomials that are related to Gamma distributions. The method is also known as Polynomial Chaos Expansion in the case of polynomial basis functions, first introduced by Norbert Wiener in 1938 (Wiener, 1938). However, note that non-polynomial functions may also be applied in SE methods, such as spherical harmonics and wavelets. The approach presented in this paper can be used for any set of orthonormal functions.

As mentioned above, Legendre are related to the standard uniform distribution U(−1,1) and Hermite polynomials to the standard normal distribution N(0,1) . In practice, however, the biological parameters often do not fit these precise restrictions. In some cases this can be repaired by applying an isoprobabilistic transformation, as we will show underneath. For example, to obtain a normally distributed random variable k with mean μ and variance σ , so k∼N(μ,σ) , starting from a θ∼N(0,1) , we need the transform

$$k=\mu +\sigma \theta ,\;\theta ∼N\left(0,1\right).$$ (7)

To obtain a uniformly distributed random variable k on the interval [a,b] , so k∼U(a,b) , from θ∼U(−1,1) , we need the transform

$$k=\left(b+a\right)/2+\left(b-a\right)\theta /2,\;\theta ∼U\left(-1,1\right).$$ (8)

A lognormally distributed random variable k∼Lognormal(μ,σ) is obtained from θ∼N(0,1) via the transformation

$$k=\mu \exp\left[\alpha \theta -\frac{\alpha^{2}}{2}\right],\;\theta ∼N\left(0,1\right),$$ (9)

Equations 7–9 show the most common transformations. Other transformations are, of course, possible as well. where $\alpha =\sqrt{\ln\left(1+\frac{\sigma^{2}}{\mu^{2}}\right)}$ .

#### 2.2.2 Multiple uncertainty parameters

Typically, biological models involve more than one random parameter, which means that the SE basis $\left\{\phi_{n}\left(\theta \right),\;n\in N^{M}\right\}$ is multivariate. Extending Equation 5 to the M-dimensional case is straightforward:

$$Y^{s}\left(\theta_{1},\ldots ,\theta_{M}\right)=\overset{N}{\underset{l_{1}=1}{\sum }}\ldots \overset{N}{\underset{l_{M}=1}{\sum }}Y\left(\lambda^{\left(l_{1}\right)},\ldots ,\lambda^{\left(l_{M}\right)}\right)u_{1}^{\left(l_{1}\right)}\ldots u_{1}^{\left(l_{M}\right)}\psi_{l_{1}}^{s}\left(\theta_{1}\right)\ldots \psi_{l_{M}}^{s}\left(\theta_{M}\right).$$ (10)

As other similar approaches, SE suffers from the curse of dimensionality (Ghanem et al., 2017; Xiu, 2007). Note from Equation 10 that the number of times the model has to be evaluated scales as $N^{M}$ , where N is the expansion order and M the number of uncertainty parameters.

#### 2.2.3 Correlated uncertainty parameters

To handle correlated parameters we propose transforming the basis functions. Here, we discuss the case of two correlated parameters; in Supplementary Material S2 we show the general case. Let $P\left(\theta_{1},\theta_{2}\right)$ be the common distribution for the uncertainty parameters $\theta_{1}$ and $\theta_{2}$ , and let $P\left(\theta_{1}\right)$ and $P\left(\theta_{2}\right)$ be the corresponding marginalised distributions. Assuming that $P\left(\theta_{1},\theta_{2}\right)\neq 0,\forall \theta_{1},\theta_{2}$ , we may write the new basis functions as (for details see Supplementary Material S2):

$$\psi_{l_{1},l_{2}}^{s}\left(\theta_{1},\theta_{2}\right)=\sqrt{\frac{P\left(\theta_{1}\right)P\left(\theta_{2}\right)}{P\left(\theta_{1},\theta_{2}\right)}}\psi_{l_{1}}^{s}\left(\theta_{1}\right)\psi_{l_{2}}^{s}\left(\theta_{2}\right),$$ (11)

where the functions $\psi_{n}^{s}$ are orthonormal with respect to P(θ) and are given by Equation 6. The expansion of a function depending on correlated parameters can be written as:

$$Y^{s}\left(\theta_{1},\theta_{2}\right)=\sqrt{\frac{P\left(\theta_{1}\right)P\left(\theta_{2}\right)}{P\left(\theta_{1},\theta_{2}\right)}}\underset{l_{1},l_{2}}{\sum }Y\left(\lambda^{\left(l_{1}\right)},\lambda^{\left(l_{2}\right)}\right)\omega_{l_{1},l_{2}}\psi_{l_{1}}^{s}\left(\theta_{1}\right)\psi_{l_{2}}^{s}\left(\theta_{2}\right),$$ (12)

with

$$\omega_{l_{1},l_{2}}=\int \sqrt{P\left(\theta_{1}\right)P\left(\theta_{2}\right)P\left(\theta_{1},\theta_{2}\right)}\psi_{l_{1}}^{s}\left(\theta_{1}\right)\psi_{l_{2}}^{s}\left(\theta_{2}\right)d\theta_{1}d\theta_{2}.$$ (13)

#### 2.2.4 Segmentation

In cases where either an expansion to high order is needed to obtain the requested approximation accuracy or the model is computationally expensive, one needs to switch to optimised or more effective methods (Le Maître and Knio, 2010; Sullivan, 2015). Models that show complex response surfaces (e.g., bifurcations) will require high order expansions to capture the inherent complexity. To overcome these problems, we present here a simple and easy to implement scheme that segments the parameter intervals into subintervals, yielding a piecewise continuous approximation of the original function. Within each of these segments we then perform a separate expansion. In this approach we have to deal with a trade-off: the number of expansions is increased, but per expansion we have a (much) lower order of expansion. Below, we argue why the second positive aspect greatly counterbalances the first negative aspect.

To determine the segments we define a scaling function $g_{m}$ with M∈N and L∈R by:

$$\begin{array}{ll} g_{m}:\left[-L,L\right] & \to I_{m}=\left[\frac{\left(2m-1\right)L}{2M+1},\frac{\left(2m+1\right)L}{2M+1}\right],m\in \left[-M,M\right]\subset Z,\\ \theta & \mapsto g_{m}\left(\theta \right)=\frac{2mL}{2M+1}+\frac{\theta }{2M+1}. \end{array}$$ (14)

This scaling function $g_{m}$ divides the interval [−L,L] into 2M+1 segments $I_{m}$ of equal length. Of course, L must be larger than or equal to any value of θ . For example, consider the case L=1 . Then, the whole interval is [−1,1] . For a segmentation granularity of M=1 , this interval is divided into three subintervals: $I_{-1}=\left[-1,-0.33\right]$ , $I_{0}=\left[-0.33,0.33\right]$ and $I_{+1}=\left[0.33,1\right]$ . The expansion of Y on any subinterval $I_{m}$ is given by:

$$Y\left(g_{m}\left(\theta \right)\right)=\overset{N}{\underset{l}{\sum }}Y\left(g_{m}\left(\lambda^{\left(l\right)}\right)\right)u_{1}^{\left(l\right)}\psi_{l}\left(\theta \right).$$ (15)

Upon a variable transformation $y=g_{m}\left(\theta \right)$ , Equation 15 becomes:

$$Y\left(y\right)=\overset{N}{\underset{l}{\sum }}Y\left(g_{m}\left(\lambda^{\left(l\right)}\right)\right)u_{1}^{\left(l\right)}\psi_{l}\left(g_{m}^{-1}\left(y\right)\right):\;y\in I_{m}.$$ (16)

After segmentation, the expansion on the interval [−L,L] as a whole is a superposition of the expansions on the subintervals:

$$Y\left(y\right)=\overset{M}{\underset{m}{\sum }}\overset{N}{\underset{l}{\sum }}Y\left(g_{m}\left(\lambda^{\left(l\right)}\right)\right)u_{1}^{\left(l\right)}\chi_{m}\left(y\right)\psi_{l}\left(g_{m}^{-1}\left(y\right)\right):\;y\in \left[-L,L\right],$$ (17)

where $\chi_{m}\left(y\right)$ is an indicator function for selecting the correct segment:

$$\chi_{m}\left(y\right)=\left\{\begin{array}{c} 1:\;y\in I_{m},\;\\ 0:\;y\notin I_{m}.\; \end{array}\right.$$ (18)

We can also define an index function to select $m^{*}\in \left[-M,M\right]$ for which $\chi_{m}\left(y\right)=1$ :

$$m^{*}=z\left(y\right)=\left\lfloor \frac{\left(2M+1\right)y}{2L}+\frac{1}{2}\right\rfloor \;\text{for}\;|y|\leq L.$$ (19)

Using both index functions we can finally write the segmented reconstruction as:

$$Y\left(y\right)=\overset{N}{\underset{l}{\sum }}Y\left(g_{z\left(y\right)}\left(\lambda^{\left(l\right)}\right)\right)u_{1}^{\left(l\right)}\psi_{l}\left(g_{z\left(y\right)}^{-1}\left(y\right)\right):\;y\in \left[-L,L\right].$$ (20)

The number of model solutions required now scales as ${\left(2M+1\right)}^{K}N^{K}$ , where K is the number of uncertainty parameters, M the segmentation granularity, and N the expansion order. The reduced accuracy by using a lower order expansion is compensated by evaluating the model more often, as a result of zooming in. Expanding up to the N -th order for p uncertainty dimensions requires solving the system $N^{p}$ times. Reconstruction requires the summation of $N^{2p}$ terms. Therefore, it is advantageous to keep N as low as possible. Normally, the reconstruction error is large for low N , but this is mitigated by segmentation. Segmentation requires to evaluate the system ${\left(2M+1\right)}^{p}N^{p}$ times, but due to segmentation N can be taken much smaller.

To illustrate this with an example, we take a system with 2 species of interest and 5 uncertainty parameters $\theta_{i}$ . The expansion order is taken as N=8 . This implies summing over $2\times 8^{10}=2,147,483,648$ terms per time point and per parameter set $\left(\theta_{1},\ldots ,\theta_{5}\right)$ . In the case of segmented expansion, we can choose a lower N , for example, N=3 with a segmentation granularity of M=1 . The number of terms to be summed over is $2\times 3^{10}=118,098$ . This is dramatically more efficient and stems from the fact that one only has to determine the segment in which the parameter set $\left(\theta_{1},\theta_{5}\right)$ falls and choose the corresponding expansion coefficients. Example II in Section 3.2 shows that segmentation may indeed be very beneficial.

#### 2.2.5 Haar wavelet expansion

Traditional SE methods are known to have difficulties with capturing discontinuous behaviour (Joel Chorin, 1974; Le Maıtre et al., 2004). Spectral convergence is only observed when solutions are sufficiently regular and continuous. Just like Fourier expansions, SE suffers from Gibbs phenomena at discontinuities, resulting in slow convergence (Le Maître and Knio, 2010). Haar wavelets have been suggested to overcome these difficulties (Le Maıtre et al., 2004; Sullivan, 2015). In contrast to global basis functions like the aforementioned polynomial systems, wavelet representations lead to localized decompositions, resulting in increased robustness at the cost of a slower convergence rate (Sullivan, 2015; Le Maître and Knio, 2010). Here, we discuss that Haar wavelets can be easily incorporated in the framework presented above and in Example IV in Section 3.4 we show how they can be applied in practice.

As mother wavelet we take

$$\psi^{W}\left(y\right)=\left\{\begin{array}{cc} 1\;\; & \text{for }0\leq y<\frac{1}{2}\\ -1\;\; & \text{for }\frac{1}{2}\leq y<1\\ 0\;\; & \text{otherwise} \end{array}\right..$$ (21)

By introducing a scaling factor j and a sliding factor k , we may construct the wavelet family

$$\psi_{j,k}^{W}\left(y\right)=2^{j/2}\psi^{W}\left(2^{j}y-k\right),\;j=0,1\;\ldots ;\;k=0,\ldots ,2^{j}-1.$$ (22)

Given the uncertainty parameter θ with its cumulative distribution function F(θ) , we define the basis functions as

$$W_{j,k}\left(\theta \right)∼\psi_{j,k}^{W}\left(F\left(\theta \right)\right).$$ (23)

By concatenating the indices j and k into one index $i\equiv 2^{j}+k$ , we may expand the meta-model Y similarly as we did in Equation 2:

$$Y\left(\theta \right)=\overset{\infty }{\underset{n=0}{\sum }}c_{n}\;W_{n}\left(\theta \right).$$ (24)

#### 2.2.6 Sensitivity analysis

In sensitivity analysis one quantifies the effects of changes in the parameters on the variability of the model response. Here, we show how our SE approach allows for sensitivity analysis in an elegant way. In the case of local sensitivity analysis, small parameter variations around a certain point in parameter space are used to determine the effect on the model output (Brian, 2013). This sensitivity is estimated via calculation of the partial derivatives of model output with respect to parameters, evaluated in that point (Ingalls, 2008). Alternatively, global sensitivity approaches do not specify a specific point in parameter space (Saltelli, 2008). For example, Sobol indices are a popular sensitivity measure as they provide a measure of global sensitivity and accurate information for most models (Ilya, 2001). Sobol indices are based on the decomposition of the variance of the output Y as a function of the contribution of the parameters (and possibly their combination), also called the ANalysis of VAriance, or ANOVA (Ilya, 2001). Thanks to the orthonormality of basis functions in SE, Sobol indices can be determined analytically from the coefficients of the SE (Sudret, 2008; Blatman and Sudret, 2010a) So, once these coefficients are known, one gets the Sobol indices nearly for free. Given the SE of output Y , the total variance of the model output is given by

$$\hat{D}=\underset{i\in I-\left\{0\right\}}{\sum }c_{i}^{2},$$ (25)

where I is the multi-index set of all variables and $c_{i}$ the expansions coefficients. The $0^{\text{th}}$ coefficient is not included as this is a constant. The partial variance is given by

$$D_{\theta_{i}}=\underset{i\in I_{\theta_{i}}}{\sum }c_{i}^{2},$$ (26)

where $I_{\theta_{i}}$ is the multi-index set of parameter $\theta_{i}$ , i.e., where the $i^{\text{th}}$ term in the multi-index is larger than 0. The Sobol indices are then given by

$$S_{\theta_{i}}=\frac{D_{\theta_{i}}}{\hat{D}},$$ (27)

In this way the relative contribution of parameter $\theta_{i}$ to the variance of the output is easily calculated.

### 2.3 Monte Carlo sampling

In the examples in Section 3.5 we compare SE with Monte Carlo sampling. For efficiency reasons we apply Quasi Monte Carlo methods using Sobol sequences, since these show a faster rate of convergence than standard sequences of pseudorandom numbers (Soboĺ, 1990). In order to test for convergence, we use the so-called blocking method. In this method the error is estimated in a straightforward manner. The quantity of interest (i.e., the model response Y ) is divided into several groups (or blocks). Then, for each block, we determine the moment of interest (e.g., mean or variance). The spread (variance) of values between blocks gives an estimate of the error. Finally, to test for convergence, we choose a threshold on the between-block variance. Note that the convergence rate of MC methods do not depend on the dimensionality of the parameter space that is being sampled, unlike SE. MC merely scales by $\sqrt{N}$ , where N is the number of samples (Christian, 2004).

### 2.4 Summary of implementation

In this section we provide an overview of the steps needed to arrive at a meta-model using SE:

1. Determine which of the model parameters is stochastic in nature and decide upon an appropriate PDF for such parameters.
2. Choose a truncation degree N .
3. Based on the PDF in the previous steps, calculate the appropriate basis functions $\phi_{n}\left(\theta \right),\;n=0,1,2\;\ldots ,N$ .
4. Determine the N×N matrix $\hat{B}$ as defined in Equation 4. For example, for Legendre polynomials $\hat{B}$ reads as

$${\hat{B}}_{n,m}=\frac{n}{\sqrt{2n+1}\sqrt{2m+1}}\delta_{nm+1}+\frac{m}{\sqrt{2n+1}\sqrt{2m+1}}\delta_{nm-1},$$ (28)

where n,m=0,1,2…,N .

For Hermitian polynomials $\hat{B}$ reads as:

$${\hat{B}}_{n,m}=\sqrt{n}\delta_{nm+1}+\sqrt{m}\delta_{nm-1}.$$ (29)

5. Calculate the eigenvalues $\lambda^{\left(l\right)},\;l=1,2,\ldots ,N$ and orthonormal eigenvectors $u^{\left(l\right)}$ .
6. Calculate $\psi_{l}^{s}\left(\theta \right)=\sum_{n=0}^{N}u_{n+1}^{\left(l\right)}\;\phi_{n}\left(\theta \right)$ .
7. Calculate $Y\left(\lambda^{\left(l\right)}\right),\;l=1,2,\ldots ,N$ by evaluating the model N times.
8. Arrive at the metamodel $Y^{s}\left(\theta \right)=\sum_{l=1}^{N}Y\left(\lambda^{\left(l\right)}\right)\;u_{1}^{\left(l\right)}\;\psi_{l}^{s}\left(\theta \right)$ .
9. Eventually, apply post-processing through, e.g., sensitivity analysis.

## 3 Results

To test the performance of the present SE approach in biological simulations, we have chosen six typical examples. Through these examples, we show how to deal with several challenges usually encountered in systems biology.

The first example has only one uncertainty parameter. Its simplicity allows comparison between the results of our approach with an exact solution.

The second example concerns a biochemical reaction network and is higher dimensional, i.e., it contains more than one uncertainty parameters. We use it to highlight the advantages of segmentation.

The third example is the glycolytic oscillator, which shows bifurcations, i.e., different dynamic behaviour for different parameter sets (Strogatz, 1994). We use it to demonstrate the power of global sensitivity analysis, which in the SE framework can be achieved without significant additional computational costs once the SE coefficients have been calculated. In addition, this example allows us to show the use of mixed expansions, since the parameter PDFs follow different distributions. This leads to a combination of different families of basis functions, thus highlighting the flexibility of the SE approach when applied to varying input uncertainties.

The fourth and fifth examples have a spatial dimension. First, we consider the Schnakenberg model which is a well-known model of pattern formation and comes with challenges such as shifts from non-patterning to patterning regions (D Murray, 2007). In this example we demonstrate the advantage of using Haar wavelets over polynomial basis functions for systems with bifurcations. Second, we study a model describing pattern formation in plants, more specifically patterning of the hairs found on top of leaves, so-called trichomes (Bouyer et al., 2008). In this model we show how to adapt the approach such that the computational costs are reduced as much as possible by carefully choosing the quantity of interest, without changing the standard set of steps.

The final example is a model of plasmid transfection, where we consider correlated parameter distributions. This model predicts the distribution of two different plasmid constructs among a population of dividing cells. Upon division the plasmids are distributed among the daughter cells according to a bivariate Poisson distribution.

### 3.1 Example I. Exponential decay: comparing performance of SE to MC and an analytical solution

For this example case, we consider the simple reaction consisting of one decaying species:


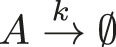
.

Its dynamics is described by $A\left(t\right)=A_{0}\exp^{-kt}$ , where $A_{0}$ is the initial concentration of A at t=0 and k the rate of decay. We test the SE method against: *1)* the exact, analytical solution and *2)* the classical Monte Carlo approach.

**FIGURE 1:**
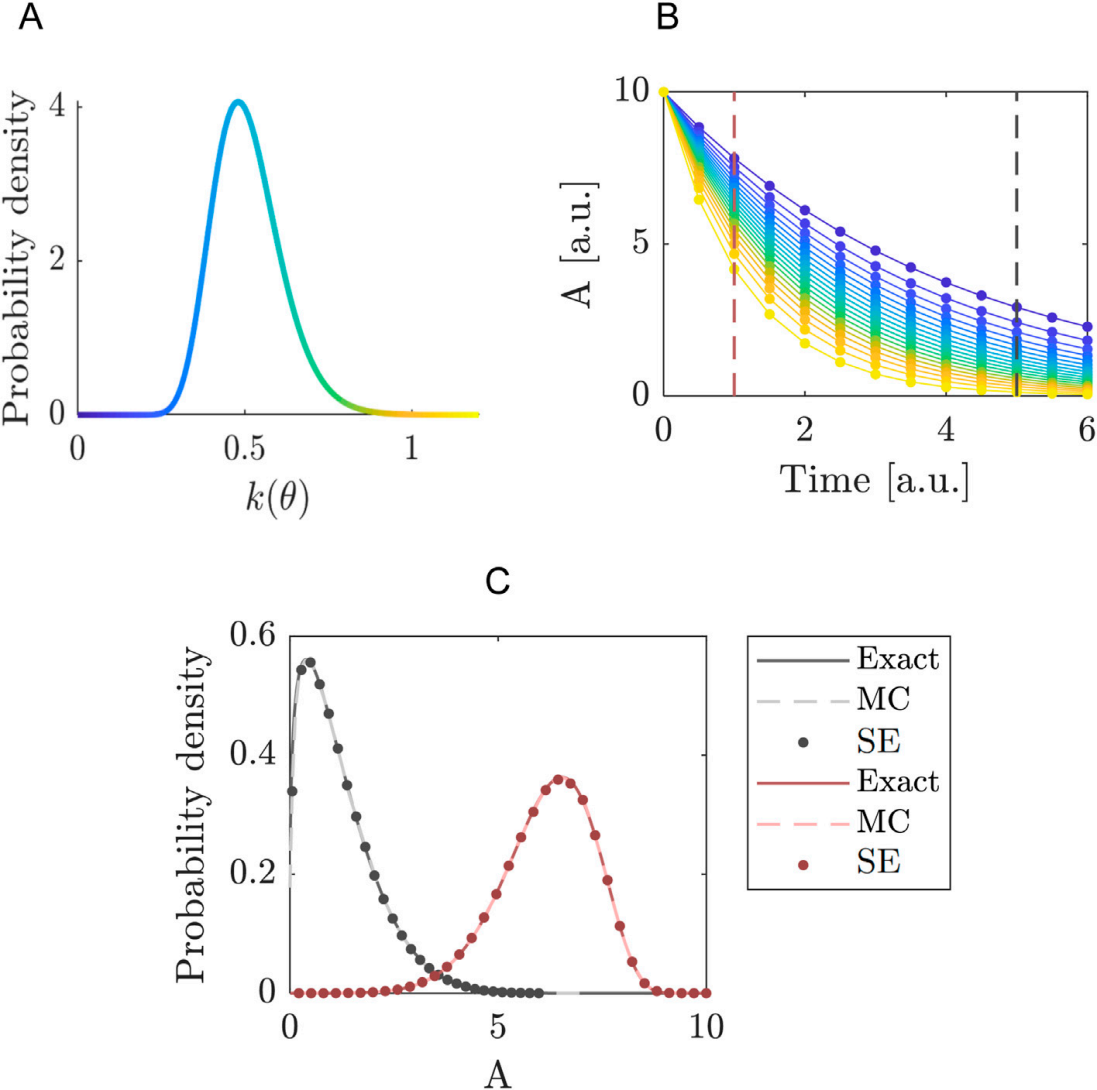
Quantifying the uncertainty propagated by the decay rate in the exponential decay model. **(A)** Probability density function of the decay rate k(θ) with μ=0.5 and σ=0.2 . The colour gradient corresponds to the value of k(θ) . **(B)** The concentration of A up to t=6 seconds. The solid lines indicate the analytical solution and the dots indicate the reconstruction using SE with Hermite basis functions and an expansion order N=5 . The colour for each of the solutions correspond to the colour of the line in A, which indicates the value of k(θ) used for each of the depicted solutions. **(C)** The dashed lines in **(B)** indicate a vertical cross-section along the model response space at t=1 for the red line, and at t=5 for the grey line, determined through three methods. First, the exact dynamics of the model (solid lines), second through MC sampling using the exact model dynamics (dashed lines) and finally, through MC sampling of the reconstructed function as obtained through SE (dots).

We assume that k is distributed according to a lognormal distribution with known mean and variance, i.e., k∼Lognormal(μ,σ) , and we choose μ=0.5,σ=0.2 . The PDF for k is shown in Figure 1 and the derivation for the exact PDF for A is given in the Supplementary Material S2.

Next, we determine how the uncertainty in k propagates through the model and affects concentration A(t) . To that end, we expand the function $A\left(t\right)=A_{0}\exp^{-kt}$ in terms of Hermitian polynomials, following the steps given in the summary and using the matrix derived for Hermitian polynomials given in Equation 29. Using Equation 9, k is transformed into a standard normal variable θ . To arrive at the meta-model $Y^{s}\left(\theta \right)$ we truncate the expansion to order N , as shown in Equation 3. Choosing N is not straightforward and will involve some experimentation. In Figure 1 we compare results for N=5 to the analytical solution for different k values. This shows that for this expansion order the reconstructed function accurately matches the analytical solution. Note that this is only based on visual inspection.

In Figure 1, we focus on the distributions of A(t=1) and A(t=5) . This is achieved by sampling the SE using a large sample set χ of reduced (i.e., standard normally distributed) variables θ , $\chi_{\mathrm{sim}}=\left\{\theta_{j},\;j=1,\ldots ,n_{\mathrm{sim}}\right\}$ . The truncated series is then evaluated onto this sample: $Y_{\mathrm{sim}}^{s}=\left\{\eta_{j}=\sum_{n=0}^{N}c_{n}\;\phi_{n}\left(\theta_{j}\right),\;j=1,\ldots ,n_{\mathrm{sim}}\right\}$ . These PDFs are obtained by kernel smoothing (Bowman and Azzalini, 1997) using a sample set with $n_{\mathrm{sim}}=10^{6}$ , drawn from the standard normal distribution with μ=0 and σ=1 . The kernel density estimator is given by

$${\hat{f}}_{Y}\left(y\right)=\frac{1}{n_{\mathrm{sim}}h}\overset{n_{\mathrm{sim}}}{\underset{j=1}{\sum }}K\left(\frac{y-\eta_{j}}{h}\right),$$ (30)

with kernel function $K\left(t\right)=\frac{1}{\sqrt{2\pi }}\exp^{-t^{2}/2}$ and bandwidth h , which is determined by Silverman’s rule of thumb (Silverman, 1986). Figure 1 shows that both MC and SE perform well (using kernel smoothing with the kernel defined in Equation 30) in reproducing.

### 3.2 Example II. Biochemical reaction network: segmentation to deal with higher dimensions

In this example we present a simple model with multiple uncertainty parameters. It allows us to illustrate the computational advantage of segmentation as explained in Section 2.2.4. The model describes the dynamics of two proteins $x_{1}$ and $x_{2}$ which bind together to form a dimer $x_{3}$ . We consider the following reactions:




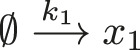

(31)






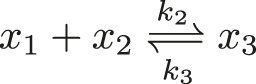

(32)






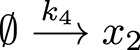

(33)






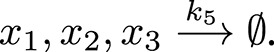

(34)



**FIGURE 2:**
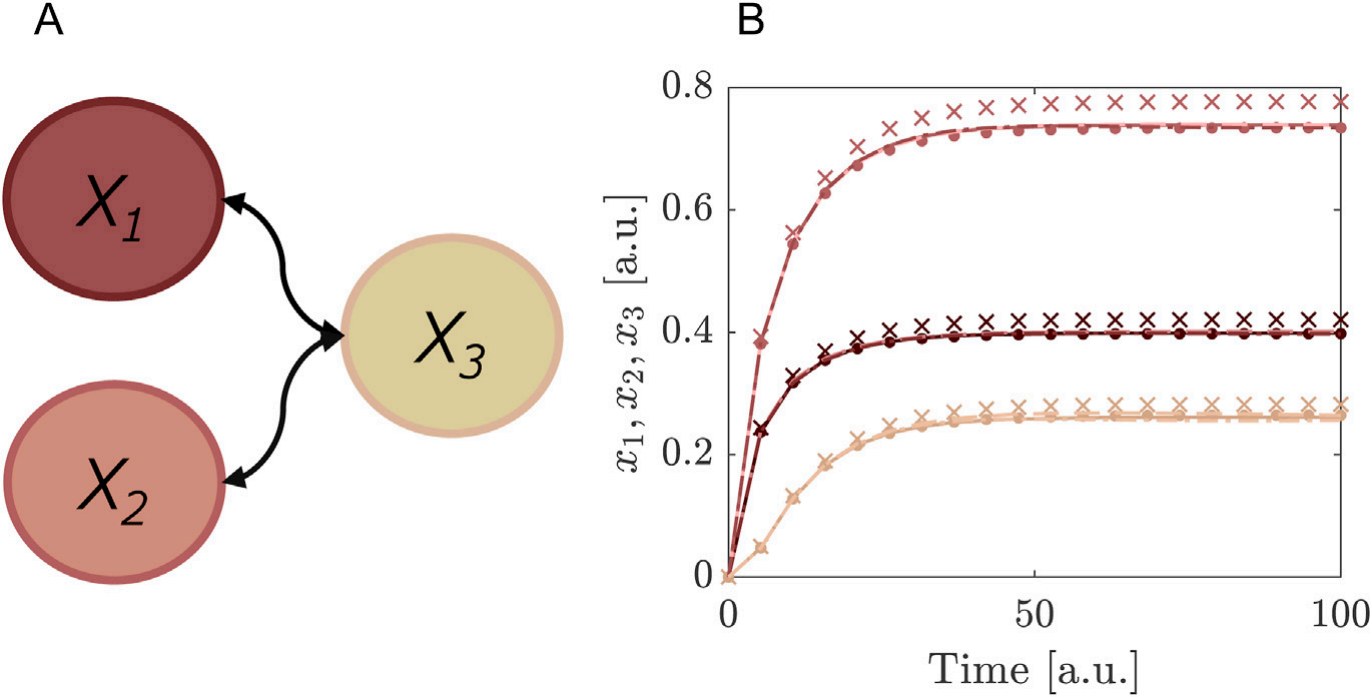
Comparing the segmented expansion with the standard non-segmented expansion. **(A)** Interaction scheme of the model in Equations 35–37. Note that $x_{1},x_{2}$ and $x_{3}$ all have the same degradation rate and $x_{1},x_{2}$ have production rates in the model but this is not indicated in the scheme. **(B)** Reconstruction of the system by a Hermitian expansion. For the segmented reconstruction we used N=2,M=1 (crosses) and N=3,M=1 (dots). For non-segmented expansion the expansion order was N=6 (dashed lines) and N=9 , (dash-dotted lines). Note that these lines overlap with the true model solution (solid lines). We used two log-normal distributions with mean and standard deviation $\mu_{1}=0.1,\sigma_{1}=0.1$ for $k_{1},k_{4},k_{5}$ and $\mu_{2}=0.4,\sigma_{2}=0.1$ for $k_{2},k_{3}$ .

In this network, the proteins $x_{1}$ and $x_{2}$ are produced at rates $k_{1}$ and $k_{4}$ . Proteins $x_{1}$ and $x_{2}$ reversibly bind to form species $x_{3}$ , with binding rate $k_{2}$ and unbinding rate $k_{3}$ . All three proteins are degraded at the rate $k_{5}$ .These interactions are visualized in a reaction scheme in Figure 2. The ODEs for thesystem given by Equations 31–34 are:

$$\dot{x_{1}}=k_{1}-k_{2}x_{1}x_{2}+k_{3}x_{3}-k_{5}x_{1}$$ (35)

$$\dot{x_{2}}=k_{4}-k_{2}x_{1}x_{2}+k_{3}x_{3}-k_{5}x_{2}$$ (36)

$$\dot{x_{3}}=k_{2}x_{1}x_{2}-k_{3}x_{3}-k_{5}x_{3}.$$ (37)

We use for the parameters $k_{1}-k_{5}$ log-normal distributions and expand the functions $x_{1}-x_{3}$ in terms of Hermitian polynomials. In Figure 2 we compare the results of the segmented expansion, see Equations 14–20, with the non-segmented expansion and the exact results. For this comparison we chose the degree of expansion and segmentation granularity such that the same number of model evaluations were required. We found that the subsequent summation to reconstruct the solutions for the differential equations improved by factors of 1,000–30,000 when using the segmented expansion. See Table 1. As mentioned in the Methods section, this improvements stems from the large reduction in the number of terms to be summed over in the segmented case compared to the non-segmented expansion.

**TABLE 1:** Benchmarking of segmented and non-segmented expansion. An overview of the number of model evaluations $N_{\lambda }$ , the number of summation terms $N_{\Sigma }$ and the time in seconds spent on summation $\left(t_{\Sigma }\right)$ , for different orders N of expansion, both segmented M=1 and non-segmented M=0 . The last column highlights the speed-up factor when segmentation is used (keeping $N_{\lambda }$ constant), i.e., N = 2, M = 1 is roughly 1,000 times faster than N = 6.

N	M	$N_{\lambda }$	$N_{\Sigma }$	$t_{\Sigma }\left[s\right]$	Speed-up factor
6	0	7,776	1.81E + 08	31.96	
9	0	59,049	1.05E + 10	5,402	
2	1	7,776	3.07E + 03	0.03	1,065
3	1	59,049	1.77E + 05	0.17	31,776

Dividing the parameter intervals into smaller sub-intervals is a relatively straightforward and simple way to circumvent huge computation times. Other, more intricate methods have been developed to tackle models with an even larger amount of parameters (Blatman and Sudret, 2010b; Xiu, 2007; Nobile et al., 2008). For example, using an adaptive algorithm that is based on classical statistical learning tools can result in a “sparse” SE, that consists of only the significant coefficients in the expansion, thereby reducing the computational cost. This method has been tested on models of stochastic finite element analysis with up to 21 parameters (Blatman and Sudret, 2010b).

### 3.3 Example III. Glycolytic oscillator: different uncertainty PDFs and global sensitivity

**FIGURE 3:**
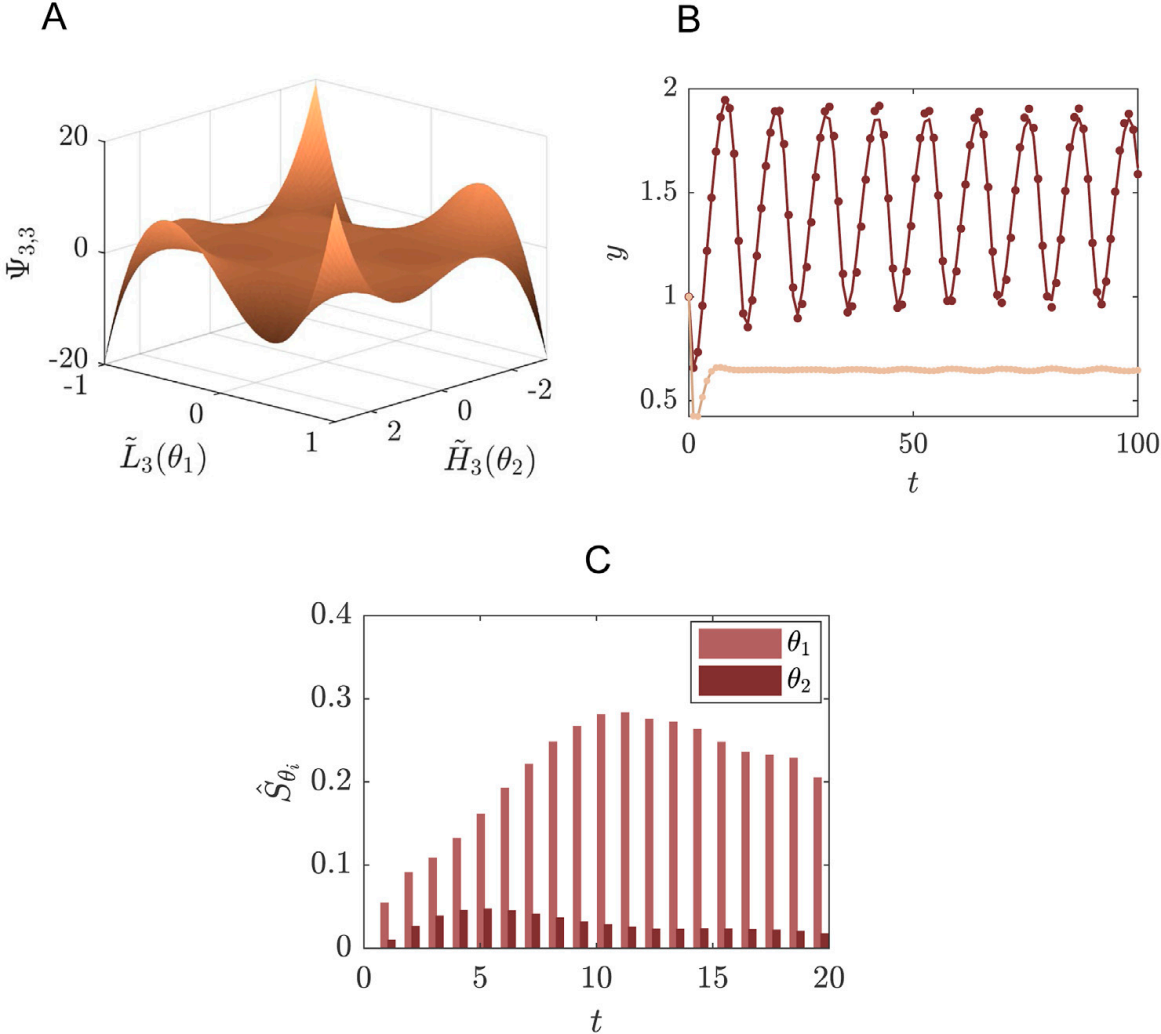
Example of a system of glycolysis as defined in Equations 38, 39 where SE is performed for the concentration of species y and for two uncertainty parameters. **(A)** Example of a multivariate polynomial, consisting of the tensor product of a 3rd Legendre polynomial of the first random variable ${\tilde{L}}_{3}\left(\theta_{1}\right)$ and the 3rd Hermite polynomial of the second random variable ${\tilde{H}}_{3}\left(\theta_{2}\right)$ . **(B)** Solutions of concentration of fructose-6-phosphate (y) in the glycolytic oscillator model for two different points in the parameter space, obtained by solving the ODEs (lines) and reconstructing via SE (dots). α=0.1,β=0.46 produces an oscillation (dark coloured) whereas α=0.5,β=0.46 gives a stable fixed point (light coloured). We used N=10 as expansion order for the Legendre and Hermite polynomials. **(C)** The first order Sobol sensitivity index ${\hat{S}}_{\theta_{i}}$ for the two random variables in the glycolytic oscillator model for the first 20 time points.

Living cells obtain energy by breaking down sugar in the biochemical process called glycolysis. In yeast cells, this glycolysis was observed to behave in an oscillatory fashion, where the concentration of various intermediates were increasing and decreasing within a period of several minutes (Hess and Boiteux, 1968). This glycolytic oscillator can be modelled as a two-component system with a negative feedback (Strogatz, 1994):

$$\dot{x}=-x+\alpha y+x^{2}y$$ (38)

$$\dot{y}=\beta -\alpha y-x^{2}y.$$ (39)

where x and y are the concentrations of ADP (adenosine diphosphate) and F6P (fructose-6-phosphate) and α,β are kinetic parameters. Depending on the values of α and β the system will be in a stable limit cycle or a stable fixed point (Strogatz, 1994). In this example, we assume α to be uniformly distributed on the interval [0.1,0.5] and β to follow a lognormal distribution with μ=0.3 and σ=0.1 . Because the two uncertainty parameters come from different distributions, we use as basis functions multivariate polynomials $\Psi_{N,M}$ which are tensor products of the univariate polynomials. In this case, Legendre polynomials are used to expand $\alpha \left(\theta_{1}\right)$ and Hermite polynomials for $\beta \left(\theta_{2}\right)$ , where $\theta_{1}∼U\left(-1,1\right)$ and $\theta_{2}∼N\left(0,1\right)$ . Note that the eigenvalues and eigenvectors used in the expansion are readily available from the matrices given in Equations 28, 29, corresponding to Legendre and Hermite polynomials, respectively. This results in a mixed polynomial for the overall expansion. As illustration, we show in Figure 3 the shape of the product of a 3rd order Legendre polynomial ${\tilde{L}}_{3}$ and a 3rd order Hermite polynomial ${\tilde{H}}_{3}$ , giving $\Psi_{3,3}={\tilde{L}}_{3}\cdot {\tilde{H}}_{3}$ .

The distributions of the uncertainty parameters were chosen such that they include the bifurcation point from stable limit cycle to the stable fixed point (Figure 3). For the purpose of this example we are interested in the concentration of y only and therefore reconstruct this model response using SE. A good approximation is obtained with a truncation degree of the SE of N=10 . This value is relatively high, due to the bifurcation in the system. However, this case shows that convergence can be reached using SE despite such challenges. It is important to note that the computational costs are still quite low.

In post-processing we may use the SE coefficients to determine the first order Sobol indices for the parameters α and β at each time point, providing a representation of the global sensitivity based on variance decomposition. The Sobol indices are readily available from the SE coefficients, as shown in Section 2.2.6. They have the advantage of being global measures of sensitivity. In Figure 3 we show the first order Sobol indices given in Equation 25–27 for the first and second random variable. They indicate the contribution to the total output variance of either $\theta_{1}$ or $\theta_{2}$ individually. Higher-order terms would give an indication of interaction effects between $\theta_{1}$ and $\theta_{2}$ , which are also readily available from the SE coefficients but are not considered here for brevity.

### 3.4 Example IV. Schnakenberg model: dealing with spatial discontinuities

In this example we introduce a spatial component. We consider the Schnackenberg model, which is one of the simplest, but yet realistic two-species system that can produce spatially oscillating solutions and therefore has become a prototype for reaction diffusion systems. The Schnakenberg model consists of the following (dimensionless) equations for the concentrations u and v of the two species (D Murray, 2007)

$$\dot{u}=\nabla^{2}u+\gamma \left(\alpha -u+u^{2}v\right),$$ (40)

$$\dot{v}=d\nabla^{2}v+\gamma \left(\beta -u^{2}v\right).$$ (41)

**FIGURE 4:**
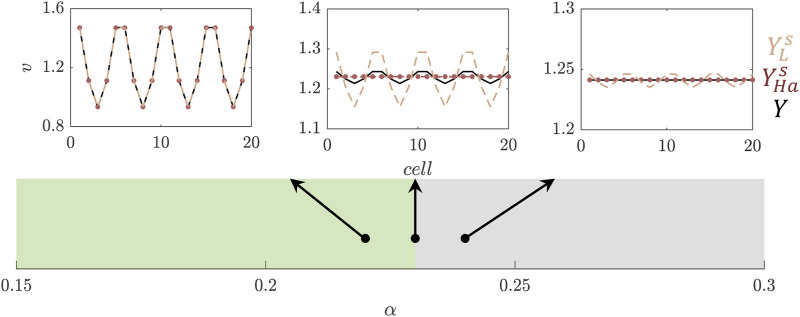
Reconstruction of the concentration of species v of the Schnakenberg model, as defined in Equation 41 at steady state, comparing Legendre and Haar wavelet expansions. We consider the patterning of v in the Schnakenberg model for α∈[0.15,0.3] , as indicated on the main *x*-axis and assuming α∼U(0.001,0.45) , while fixing β=1,γ=5 and d=20 . The colour along this axis indicates the region in the 1D-parameter space whether a stable pattern will form (i.e., α is inside the Turing Space (TS), indicated by green colouring), or a homogeneous spatial distribution of v (grey colour). We highlight three examples of the patterns formed for different values of α , one inside the TS (left inset, α=0.22 ), one close to the boundary (middle inset, α=0.23 ) and one outside the TS (right inset, α=0.24 ). Within these examples we compare the true solution Y (solid black line) to the reconstructed function of v by SE in terms of Legendre polynomials $Y_{L}^{s}$ by a segmented expansion with polynomial order N=18 and segmentation granularity M=3 (dashed line), and Haar wavelets $Y_{Ha}^{s}$ (dots), using the first 128 wavelets in the expansion, i.e., N=6 resolution levels.

α,β are reaction rates, γ a scale parameter and d the ratio of diffusion constants between the species u and v . The species u is auto-catalytically produced by the $u^{2}v$ term in Equation 40, whereby species v is consumed. There are certain combinations of the parameters α,β,γ and d for which the system will exhibit a stable pattern (Liu et al., 2013); this region of parameter space is called the Turing space (TS). For illustrational purposes we limit the number of uncertainty parameters to one: the parameter α , fixing the other parameters at β=1,γ=5 and d=20 . We assume α to be distributed as α∼U(0.001,0.45) and determine the TS for a range of α (Figure 4) using linear stability analysis [for details see (D Murray, 2007; Liu et al., 2013)]. To that end, the model is simulated on a 1D grid of 20 cells. We focus on the concentration of species v at steady state and compare expansions in terms of Legendre polynomials and of Haar wavelets.

SE using polynomial basis functions is known for being inaccurate in regions that contain discontinuities (Le Maître and Knio, 2010; Le Maıtre et al., 2004; Joel Chorin, 1974). In this example, the lack of convergence in SE can be seen along the boundary of the patterning space (TS) in Figure 4, where the expansion by Legendre polynomials is indicated with the dashed lines. For the reconstruction of concentration v in terms of Legendre polynomials, we used a segmented expansion with N=18 and a segmentation granularity of M=3 , leading to a total of 126 model evaluations used in the expansion. To show that Haar wavelets perform much better in such a region, we also consider an expansion in terms of Haar wavelets (Equations 21–24). As resolution level we take N=6 , which means a total of $N_{w}=128$ wavelets are used in the expansion. In Figure 4 the performances of Legendre polynomials and Haar wavelets are compared in the vicinity of α=0.23 (middle inset, Figure 4), showing that the Haar-wavelets provide an improvement in accuracy at the bifurcation point, while using the same number of model evaluations (i.e., the same amount of information and computational cost) for the expansion.

### 3.5 Example V. Trichome patterning: dealing with spatial discontinuities

**FIGURE 5:**
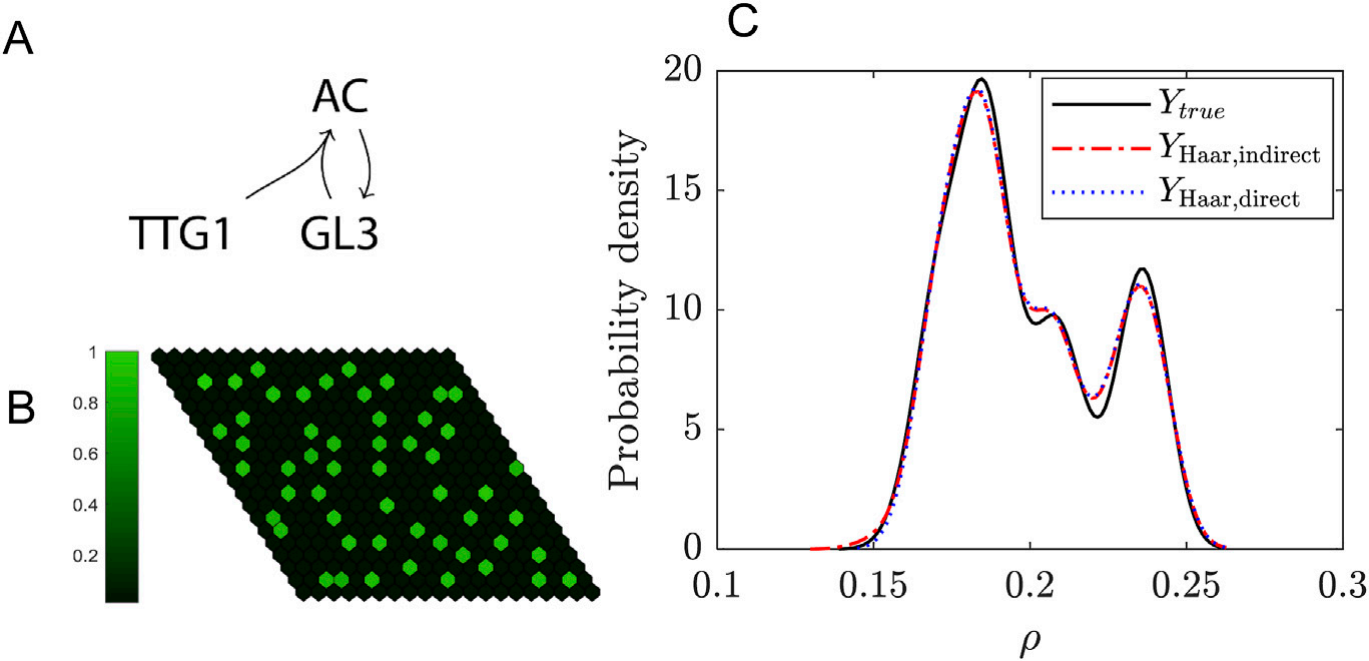
Uncertainty quantification for the trichome system. **(A)** Schematic of the model. **(B)** Example of a simulation of the model on a 20-by-20 grid. The colorbar indicates the levels of the activating complex (AC) in the cells relative to the maximum. **(C)** Probability density function of the trichome density in the Turing Space using either the indirect (dashed red line) or direct expansion (dotted blue line) method and for comparison the solution of the real model (solid black line). A resolution level of 3 (i.e., a total of 16 wavelets) has been used for the expansion.

As an extra example of pattern formation we consider a model that describes trichomes. Trichomes are hairs found on the epidermal layer of leaves. In *Arabidopsis Thaliana* these trichomes form a regular pattern, where each trichome is separated by around three to four epidermal cells (Hülskamp, 2004). The model studied here consists of three proteins and their interactions which can explain features of trichome patterning (Bouyer et al., 2008; Pesch and Hülskamp, 2004). Protein transpararant testa glabra1 (TTG1) binds to the transcription factor glabra3 (GL3) which together form a trichome-promoting complex, called the activating complex (AC) (Bouyer et al., 2008). Experimental data suggests that TTG1 is depleted from cells neighbouring a trichome (Bouyer et al., 2008). For this reason the interaction between TTG1 and GL3 is modelled in a substrate-depletion form (Figure 5), where TTG1 acts as a substrate for the formation of AC (Bouyer et al., 2008). After non-dimensionalisation this model consists of four parameters, none of which have been experimentally determined, highlighting the substantial amount of uncertainty within this model (Pesch and Hülskamp, 2009; Scheres, 2000). Here, we examine the propagation of uncertainty in the parameters to the predicted pattern.

The trichome patterning is described by the following set of coupled ODEs (Bouyer et al., 2008):

$$\dot{{TTG1}_{j}}=\alpha -\lambda {TTG1}_{j}-{TTG1}_{j}{GL3}_{j}+\delta \hat{L}{TTG1}_{j}$$ (42)

$$\dot{{GL3}_{j}}=\beta {AC}_{j}^{2}-{GL3}_{j}-{TTG1}_{j}{GL3}_{j}$$ (43)

$$\dot{{AC}_{j}}={TTG1}_{j}{GL3}_{j}-{AC}_{j},$$ (44)

where α,λ,δ and β are parameters in the model and $\hat{L}$ describes the coupling between the cells. The subscript j indicates the $j^{\text{th}}$ cell. We solve Equations 42–44 for 400 cells, grouped on a hexagonal grid of 20 by 20 cells.

In this example we focus on the parameter α , the basal production for TTG1. We assume this parameter to be uniformly distributed on the interval [0.4,0.9] . We are interested in the number of trichomes that are predicted by the model, therefore we consider the trichome density ρ (total number of trichomes divided by the total number of cells in the simulated tissue) as the model response of interest. The number of trichomes is determined by simulating the system until steady state is reached and counting the number of cells for which the concentration of AC exceeds a threshold. The amount of AC is considered to be an indicator for trichome cell fate in plants. However, the biological threshold for this is unknown. We set this threshold to the half-maximum of AC in the system. This leads to the following description of trichome density:

$$\rho =\frac{\left|T\right|}{N},$$ (45)

where T is the set of cells which exceed the AC threshold, J is the set of all cells on the grid, N is the total number of cells and |T| is the cardinality of T .

Our present goal is to study the uncertainty in ρ as a result of the uncertainty in α . To this end, we employ two different approaches. For both approaches we first transform α to a standard uniform variable by $\alpha =T^{-1}\left(\theta \right)$ , using the transform function for a uniform variable given in Equation 8. The first approach, referred to as the indirect approach, is the same as used in Example IV in Section 3.4. To reconstruct the concentration at steady state for all cells, we expand the concentration of AC in terms of Haar wavelets. From the result we may determine ρ . In this process we discriminate between cases where there is a pattern and where there is no pattern. Through linear stability analysis we determine beforehand whether a pattern will form or not, i.e., whether the chosen parameter set is in the Turing Space (TS) (D Murray, 2007). For a certain realisation θ we can determine ρ (Equation 45) by:

$$\rho \left(\theta \right)=\left\{\begin{array}{cc} \frac{\left|T^{s}\right|}{N}\; & \text{if}\;\theta \in TS\\ 0\; & \text{if}\;\theta \notin TS \end{array}\right.,$$ (46)

where $T^{s}$ is the set of trichomes as determined from the reconstructed AC concentration profile.

Our second, direct approach is to directly reconstruct ρ as

$$\rho^{s}\left(\theta \right)=\overset{N}{\underset{l=1}{\sum }}Y\left(\lambda^{\left(l\right)}\right)\;u_{1}^{\left(l\right)}\;\psi_{l}^{s}\left(\theta \right).$$ (47)

Similarly, as we did for ρ(θ) we can define $Y\left(\lambda^{\left(l\right)}\right)$ as

$$Y\left(\lambda^{\left(l\right)}\right)=\left\{\begin{array}{cc} \frac{\left|T\right|}{N}\; & \text{if}\;\lambda^{\left(l\right)}\in TS\\ 0\; & \text{if}\;\lambda^{\left(l\right)}\notin TS \end{array}\right..$$ (48)

In other words, we only solve the system and determine the trichome density if the parameter set falls within the Turing space. This lends robustness to the SE for the non-smooth parts of the function Y(θ) and at the same time limits the amount of simulations to be performed, as the non-patterning parameter combinations need not be solved for.

One of the nice features of this Example is that it illustrates there are multiple ways in which the uncertainty in the output can be captured: first, an indirect method (Equation 46) where the model output consists of concentration profiles from which the pattern features have to be extracted in post-processing, and second, by taking the density as model output (Equations 47, 48). In Figure 5 results for the indirect and direct approaches are compared. We conclude that both have similar levels of accuracy and in both cases the expansions converge to the real solution at resolution level N = 6, which means a summation of 128 wavelets. The PDF in Figure 5 is constructed using $10^{3}$ samples which costs 4.7 s for the wavelet reconstruction as opposed to 80.9 s for solving the full model a 1,000 times in an MC approach.

### 3.6 Example VI. Plasmid transfection: dealing with correlated parameters

**FIGURE 6:**
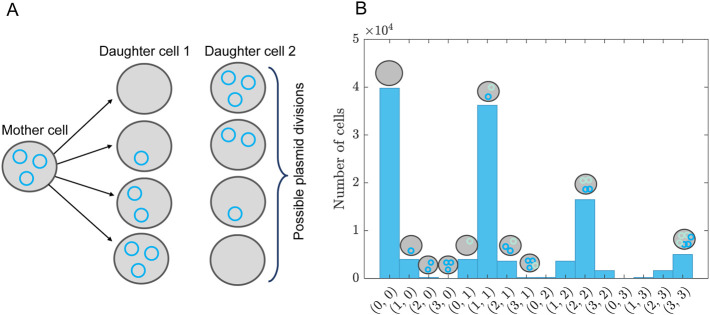
Plasmid distribution model. **(A)** Schematic overview of plasmid distribution upon cell division, showing all possible combinations of how to divide three plasmids among two daughter cells. This is for a single type of plasmid. The probability of finding these combinations is given by Equation 56. **(B)** Distribution of plasmid combinations for two types of plasmids, assuming a bivariate Poisson distribution given by Equation 59 with λ=1 and ξ=0.9 and a population of 100.000 cells. The *x*-axis indicates the number of type n and m plasmids as a tuple (n,m) , visualized in a few example cells above the bars, where the light-blue circles indicate plasmids of type n and the dark blue plasmids of type m . The *y*-axis shows the number of cells that have a certain amount of n,m -type plasmids.

It can happen that in parameter space a structure occurs, i.e., that the multivariate joined probability cannot be written as a product of univariate distributions. In this section we exemplify how to handle such a case. As an example we choose the transfection of mammalian cells (e.g., Human embryonic kidney cells, HEK293) with two plasmids: plasmid $pl_{1}$ with the construct for induction (e.g., through chemical or optogenetics (Yoshida and Sato, 2009)), and plasmid $pl_{2}$ with the reporter construct. In short, one of the plasmids is required to activate the cell $\left(pl_{1}\right)$ , the other is used for read-out $\left(pl_{2}\right)$ . In the part below we describe a model that predicts the distribution of both types of plasmids among a population of cells, assuming that the plasmids will have to be distributed among daughter cells upon division (Figure 6A). We assume that this distribution of plasmids is accurately described by a bivariate Poisson distribution (example given in Figure 6B), as we will argue below.

Denoting by n the number of $pl_{1}$ plasmids and by m the number of $pl_{2}$ plasmids in a specific cell, the corresponding reaction scheme reads:




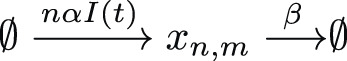







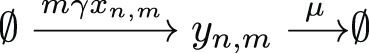







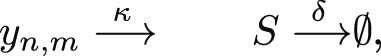

where $x_{n,m}$ and $y_{n,m}$ are the concentrations of molecule x and y, resp., in a cell with plasmid composition nm . S is the concentration of the reporter molecule in the bulk (i.e., the read-out produced by the reporter construct of plasmid $pl_{2}$ ). α and γ are the corresponding production rates per plasmid and β , μ are the degradation rates. I(t) is the external induction signal (i.e., the signal required to activate the plasmid $pl_{1}$ ). In what follows we will set I(t)=1 for t≥0 .

For sake of clarity we keep the system simple and ignore all complicating effects, such as gene expression noise, maturation of the reporter construct (e.g., GFP). However, for transient transfections of the mammalian cells, we need to take into account the distribution of plasmids among the cells, which is not constant due to cell division. The set of Ordinary Differential Equations describing the reactions given by Equations 49–51 read:

$${\dot{x}}_{n,m}=n\alpha I\left(t\right)-\beta x_{n,m}$$ (52)

$${\dot{y}}_{n,m}=\frac{m\gamma x_{n,m}}{1+\eta x_{n,m}}-\left(\mu +\kappa \right)y_{n,m}$$ (53)

$${\dot{Q}}_{n,m}=\omega \left(1-\frac{\|Q{\|}_{1}}{N_{\text{max}}}\right)\underset{p,q}{\sum }L_{n,m}^{p,q}Q_{p,q}$$ (54)

$$\dot{S}=v\kappa \underset{n,m}{\sum }Q_{n,m}y_{n,m}-\delta S.$$ (55)

The parameter η captures the saturation of the expression capacity of the cells. We set $\eta =0.2\;V^{-1}$ throughout the calculations. V is the unit of the volume of the cells, e.g., $V=\mu m^{3}$ . $Q_{n,m}$ is the number of cells that have n,m plasmids which are growing by ω . To follow the partition of plasmids upon division, the tensor L is introduced and described in Equations 57, 58 below. $\|Q{\|}_{1}=\sum_{n,m}Q_{n,m}=N_{\text{tot}}$ which is the total number of cells at time t and $N_{\text{max}}$ is the maximum number of cells due to environmental constraints. The growth rate is set to $\omega =0.034\;h^{-1}$ based on a doubling time of 20 h as being measured for HEK293T cells (Tan et al., 2021; Zhang et al., 2024). S is the reporter [e.g., SEAP (Berger et al., 1988)] accumulated in the bulk due to secretion by the cells with rate κ . For non-secreted reporters (e.g., YFP) for single cell measurements, κ is set to zero. v is a volume correction factor, set to $v=10^{-8}$ .

We assume that the rate of dilution of the plasmids corresponds to the growth rate of the cells, i.e., plasmids are lost upon division. In reality, the plasmids are also degraded, but we assume the time scale of this degradation is much longer than the experimental duration and can therefore be ignored. To model the partition of plasmids upon cell division we assume that any partition of the number of plasmids is possible, see, for example, Figure 6A. The probability of finding a certain partition is given by the binomial distribution

Pnk=θnkkn1−qk−nqn, (56)

where n is the number of plasmids in the mother cell, k the number of plasmids in one of the daughter cells and q the probability, which for equal cell division is q=0.5 . $\theta_{n}^{k}$ is the discrete unit step function with $\theta_{n}^{k}=1$ for k≥n and $\theta_{n}^{k}=0$ else. We can write the rate of change in the pools of cells as

$${\dot{Q}}_{n,m}=2\underset{k,p}{\sum }P_{n}^{k}P_{m}^{p}Q_{k,p}-Q_{n,m}=\underset{k,p}{\sum }\left(2P_{n}^{k}P_{m}^{p}-\delta_{n}^{k}\delta_{m}^{p}\right)Q_{k,p}.$$ (57)

$\delta_{n}^{k}=1$ for k=n and $\delta_{n}^{k}=0$ else. From this, we can define the tensor used in Equation 54:

$${\dot{Q}}_{n,m}=\Omega (2\underset{k,p}{\sum }P_{n}^{k}P_{m}^{p}Q_{k,p}-Q_{n,m})=\Omega \underset{k,p}{\sum }\left(2P_{n}^{k}P_{m}^{p}-\delta_{n}^{k}\delta_{m}^{p}\right)Q_{k,p}.$$ (58)

We treat the plasmid uptake as a Poisson process, i.e., the number of plasmids inside a cell is Poisson distributed. Assuming that the mean number of plasmid taken up is the same for $pl_{1}$ and $pl_{2}$ and a correlation exists between the uptake of two plasmids, the distribution is given by (Berkhout and Plug, 2004):

$$P\left(n,m\right)=\frac{{\left(\lambda -\xi \right)}^{m}}{{\left(\lambda -\xi \right)}^{n}}\frac{{\left(-1\right)}^{n}\xi^{n}}{n!m!}U\left(-n,1-n+m,-\frac{{\left(\lambda -\xi \right)}^{2}}{\xi }\right)e^{-2\lambda +\xi },$$ (59)

where U is Kummer’s confluent hypergeometric function (Olver, 2010), λ is the mean number of plasmids taken up, and 0≤ξ≤λ is the correlation parameter. Note that for ξ→0 we find:

$$\underset{\xi \to 0}{\lim}P\left(n,m\right)=P\left(n\right)P\left(m\right),\;\text{with: }P\left(x\right)=\frac{\lambda^{x}}{x!}e^{-\lambda }.$$ (60)

The correlation between n and m reads:

$$corr\left(n,m\right)=\frac{cov\left(n,m\right)}{\sqrt{var\left(n\right)var\left(m\right)}}=\frac{\xi }{\lambda }.$$ (61)

**FIGURE 7:**
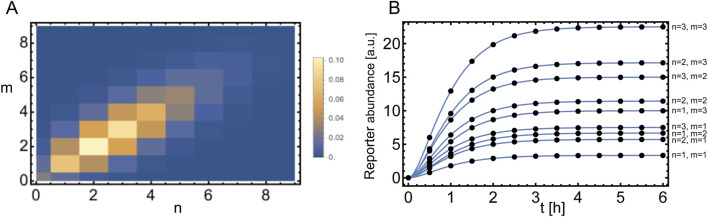
Expansion with respect to a bivariate Poisson distribution. **(A)** Bivariate Poisson distribution given by Equation 59 with λ=3 and ξ=2.4 . **(B)** Comparison of the exact solution for $y_{n,m}\left(t\right)$ given by Equation 53 (solid lines) to the spectral expansion with N=6 (dots). Parameters: $\alpha =2\;{\left(Vh\right)}^{-1}$ , $\beta =2\;h^{-1}$ , $\gamma =6\;h^{-1}$ , $\eta =0.5\;V^{-1}$ , $\mu =1.5\;h^{-1}$ , $\kappa =0\;h^{-1}$ .

In Figure 7A we show P(n,m) for λ=3 and ξ=2.4 . The asymmetry due to the correlation corr(n,m)=0.8 can be clearly seen. The orthonormal basis function $\phi_{p}\left(n\right)$ with respect to the Poisson distribution read (Ogura, 1972):

ϕpn=F02−p,−n;;−1λλpp!. (62)

F02 is a generalised hypergeometric function (Olver, 2010). The matrix $\hat{B}$ defined in Equation 4 can be calculated analytically and is given by:

$${\hat{B}}_{n,m}=\left(n+\lambda \right)\delta_{nm}-\sqrt{n\lambda }\delta_{n-1m}-\sqrt{m\lambda }\delta_{nm-1}.$$ (63)

Note the dependence on the parameter λ , in contrast to the matrices given in Equations 28, 29.

**FIGURE 8:**
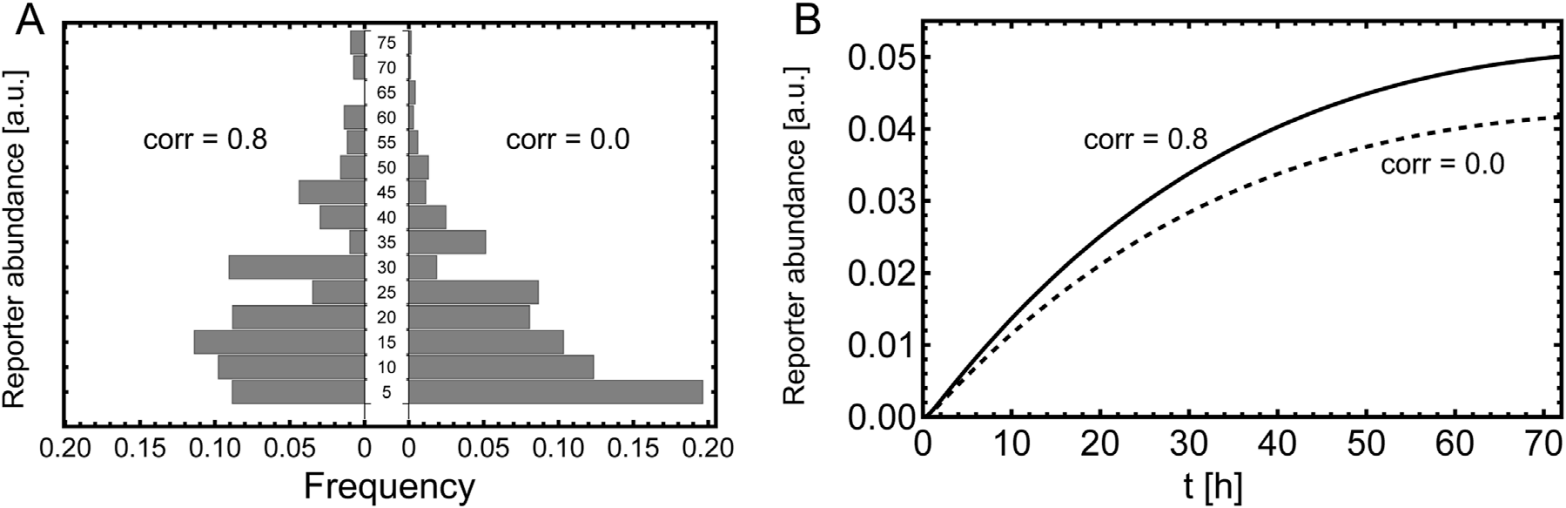
Measurement of the reporter. Comparison of correlated and un-correlated plasmid uptake. **(A)** Histogram of the single cell readout, measured 6 h after induction. The parameters are the same as stated in Figure 7 and the histogram is calculated according Equation 64 with g=5 **(B)** Reporter abundance in the bulk measured for 3 days. The parameters are the same as in A, besides $\kappa =3\;h^{-1}$ , $\delta =0.01\;h^{-1}$ and $v=10^{-8}$ . We used for the calculations the expansion of $y_{n,m}$ with N=6 .

We first consider a system in which the mammalian cells do not secrete the reporter molecule, e.g., YFP. In this case we set κ=0 , and consequently S(t)=0,∀t . In Figure 7B we show $y_{n,m}\left(t\right)$ for different plasmid compositions and compare the expansion of $y_{n,m}\left(t\right)$ , using Equations 11–13 and Equations 59–63, of the order N=6 (dots) to the exact results (solid lines). A question of interest is what is the measured distribution of fluorescence intensities of the mammalian cells. The distribution of reporter molecule abundance is given by:

$$P\left(z,t\right)=\overset{\infty }{\underset{n,m}{\sum }}\delta_{z,\chi_{n,m}\left(t\right)}\frac{Q_{n,m}\left(t\right)}{\|Q{\|}_{1}},$$ (64)

where $\chi_{n,m}\left(t\right)$ is a binning function: $\chi_{n,m}\left(t\right)=g\left\lfloor y_{n,m}\left(t\right)/g\right\rfloor$ (other binning methods are also possible, of course). Note that for sake of simplicity we ignore the transient phase after cell division in which the dynamic of $y_{n,m}$ adopts to the new, reduced plasmid composition. This is also a reasonable simplification considering that the steady state of $y_{n,m}$ is roughly reached after 4 h , as can be seen in Figure 7B, in contrast to a cell doubling time of 20 h. In Figure 8A we show the distribution P(z,t=50) for g=5 for ξ=2.4 (left) and ξ=0 (right); we used the expansion with N=6 for the calculations in Equation 64.

Next, we consider a segregated reporter, e.g., SEAP. In this case the reporter abundance in the bulk is measured instead of single cell measurements. The temporal evolution of the bulk reporter is governed by Equations 52–55. In Figure 8B one can see that the dynamics of the reporter is clearly different due to correlation of the plasmid uptake.

## 4 Discussion and conclusion

In this paper we have presented an efficient and widely-applicable version of spectral expansion to quantify the effect of parameter uncertainty on model outcomes. The present scheme is based on non-intrusive spectral projection and makes use of expansions in terms of some orthonormal set of basis functions., e.g.,,polynomial functions or wavelets. The orthonormal properties of those basis functions are utilized to develop a novel scheme to determine the expansion coefficients. It has the attractive property that it is computationally very fast. The scheme is similar to the Golub-Welsh algorithm known from Gaussian quadrature (Golub and Welsch, 1969). In the latter procedure the roots of the polynomials used play an essential role (Bruno, 2007). In our new scheme this role is played by the eigenvalues of a matrix, which can be calculated very fast. Our method does not require modification of the model equations. This is in general the main advantage of non-intrusive methods: there is no need to recast the model into a probabilistic framework. Instead, the random behaviour of parameters is accounted for through a set of deterministic simulations of the process for a restricted number of parameter values. These values are chosen such that they reflect the uncertainties in the parameters. To test the performance of our method we applied it to a number of different models (Example I) a model of exponential decay (Example II) a biochemical reaction network (Example III) the glycolytic oscillator (Example IV) the Schnakenberg model, and (Example V) a trichome model. Examples IV and V deal with spatial pattern formation, and, finally, Example VI illustrates SE for correlated parameters. For each test case, the results of the SE are compared to, if available, analytical solutions and/or and Monte Carlo simulations. In these comparisons we mostly focus on the accuracy of SE. Although the computational advantage is an important reason for using SE techniques, we do not focus on that aspect since it has already been extensively explored elsewhere; see, for example, (Zein et al., 2013; Bruno, 2007; Fajraoui et al., 2017).

The accuracy of the reconstruction by SE depends on the choice of expansion order and the appropriate choice of basis functions. While the latter choice is determined by the PDFs of the input parameters, the choice of expansion order has to be chosen by the user. For example, in Examples I and III we chose N=5 and N=10 , respectively. These choices were based on careful observation of the convergence properties of the method.

In some cases the expansion order has to be chosen prohibitively large. For such a situation we propose an extended approach that segments the parameter interval into subintervals, essentially zooming in on these sub-intervals such that a lower expansion order can be used in each sub-interval. In Example II we have shown that this segmentation approach can greatly reduce the computational costs, thus providing a way to circumvent the curse of dimensionality. Such adaptations are nearly always required in high-dimensional cases and segmentation is a relatively simple and straight-forward method to tackle dimensionality problems yielding a piecewise continuous approximation to the original function. It is an alternative for so-called sparse SE methods, that utilise only a small subset of the basis functions in order to limit the amount of model evaluations (Blatman and Sudret, 2010b; Xiu, 2007; Nobile et al., 2008).

Convergence of the SE may be poor in regions of the parameter space around a bifurcation (Ghanem et al., 2017), due to the use of smooth basis functions to represent non-smooth model behaviour. This effect is often illustrated by the Gibbs phenomenon in Fourier expansions, where the spectral basis consisting of smooth sine and cosine functions is not suitable, giving rise to slow and even lack of correct convergence. Since smooth functions like the Hermite and Legendre polynomials will fail to describe steep or discontinuous solutions, we explored the use of Haar wavelets. Wavelets naturally allow localised decompositions and this leads to more robust behaviour (Le Maıtre et al., 2004). We show in Example IV (Schnakenberg model) the advantages of using Haar wavelets over polynomials by focusing on the region in parameter space where the system jumps from spatially heterogeneous to spatially homogeneous dynamics. Although around this bifurcation point an expansion in terms of Haar wavelets also turned out to show slow convergence, the accuracy of the expansion is much better than when Legendre polynomials are used. In the vicinity of bifurcations Haar wavelets thus provide a useful tool for biological systems which feature discontinuities. In Example V (trichome pattern formation) we have highlighted the flexibility of the method: some quantities, e.g., the scalar quantity of trichome density, can either be directly expanded or indirectly. By making use of that adaptability the number of model evaluations can be reduced while the level of accuracy is maintained. Finally, in Example VI we illustrate how to handle correlated parameters by means of correlated plasmid uptake by mammalian cells. We show how single-cell or bulk readout can be calculated using SE.

Overall, the approach presented here consists of a number of easy-to-implement steps and is applicable to a great variety of systems that would be computationally costly when analysed in the context of uncertainty quantification in the usual way. We therefore believe that this approach could provide a valuable asset for the toolkit of computational systems biology.

## Data Availability

The code is publicly available at: https://github.com/AnnaDeneer/SpectralExpansion.
